# An Efficient System for Eye Movement Desensitization and Reprocessing (EMDR) Therapy: A Pilot Study

**DOI:** 10.3390/healthcare10010133

**Published:** 2022-01-10

**Authors:** Nicolae Goga, Costin-Anton Boiangiu, Andrei Vasilateanu, Alexandru-Filip Popovici, Marius-Valentin Drăgoi, Ramona Popovici, Ionatan Octavian Gancea, Mihail Cristian Pîrlog, Ramona Cristina Popa, Anton Hadăr

**Affiliations:** 1 Faculty of Engineering in Foreign Languages, University Politehnica of Bucharest, Splaiul Independentei 313, 060032 Bucharest, Romania; n.goga@rug.nl (N.G.); andrei.vasilateanu@upb.ro (A.V.); marius.valentin.dragoi@gmail.com (M.-V.D.); ramona.popovici@drd.unibuc.ro (R.P.); popa.ramona91@yahoo.com (R.C.P.); 2 Faculty of Automatics and Computer Science, University Politehnica of Bucharest, Splaiul Independentei 313, 060032 Bucharest, Romania; costin.boiangiu@cs.pub.ro (C.-A.B.); ganceatoni@gmail.com (I.O.G.); 3 Faculty of Psychology and Educational Sciences, University of Bucharest, 90 Panduri Street, 050657 Bucharest, Romania; 4 Department of Sociology, University of Medicine and Pharmacy of Craiova, Petru Rares 2, 200349 Craiova, Romania; mihai.pirlog@gmail.com; 5 Faculty of Industrial Engineering and Robotics, University Politehnica of Bucharest, Splaiul Independentei 313, 060032 Bucharest, Romania; anton.hadar@upb.ro

**Keywords:** EMDR, virtual assistant, cyber-physical system

## Abstract

In this paper, we describe an actuator-based EMDR (eye movement desensitization and reprocessing) virtual assistant system that can be used for the treatment of participants with traumatic memories. EMDR is a psychological therapy designed to treat emotional distress caused by a traumatic event from the past, most frequently in post-traumatic stress disorder treatment. We implemented a system based on video, tactile, and audio actuators which includes an artificial intelligence chatbot, making the system capable of acting autonomously. We tested the system on a sample of 31 participants. Our results showed the efficiency of the EMDR virtual assistant system in reducing anxiety, distress, and negative cognitions and emotions associated with the traumatic memory. There are no such systems reported in the existing literature. Through the present research, we fill this gap by describing a system that can be used by patients with traumatic memories.

## 1. Introduction

Eye movement desensitization and reprocessing (EMDR) is an eight-phase psychological therapy designed to treat emotional distress caused by traumatic events from the past, most frequently in post-traumatic stress disorder (PTSD). PTSD is a serious disorder that can be developed by people after exposure to severe stressors that generate a series of symptoms such as avoidance of reminders of the event, hyperarousal, or vivid flashbacks [1]. During the therapy, the clinician uses diverse bilateral stimuli such as a light that changes positions, the alternation of sounds between speakers, or the vibration of devices held by the patient in their hands, in order to help the client process a distressing experience.

Although studies have shown that EMDR is an efficient method of intervention for different mental conditions, there are still controversies regarding the working mechanism. The studies so far on EMDR efficiency and the role of eye movement have captured variable results [2], which makes the emergence of more rigorous research an imperative [3]. In this context, the tools for self-administration of EMDR therapy can facilitate the understanding of the role of eye movement in EMDR therapy.

A study from 2012 [4] found that intervention through EMDR helped the participants in coping with a psychotic disorder and PTSD. Also, another study showed that EMDR had better results in treating specific symptoms than other types of therapies [5]. A more recent meta-analysis showed that EMDR treatment is efficient in reducing PTSD symptoms [6] and other mental health problems such as anxiety and depression [7,8]. Therefore, EMDR therapy has proven to be effective in treating trauma and PTSD. Moreover, EMDR may be effective for any psychological disorder that can be linked with trauma or adverse life events [9].

In the light of this information, an actuator-based virtual assistant trained to perform EMDR therapy could be a useful tool for psychologists in the process of treating their patients. It can also become a tool used by the general population for the treatment of minor psychological disorders linked with trauma. The artificial intelligence engine chatbot developed for the presented system was based on the eight essential phases of EMDR described by Francine Shapiro [10]:

1. The first phase involves the investigation of the client’s past in order to design the treatment plan. This includes the evaluation of all clinical aspects (symptoms, unhealthy behaviors, and characteristics) and the selection made by the therapist of the issues that need to be addressed (events that affect the emotional life, dysfunction triggers, and new positive behaviors/attitudes).

2. In the preparation phase, the therapist explains the basic theory of EMDR and the intervention procedures with the aim of setting the expectation of the outcome, which also includes the presentation of the possible disturbances that could appear. The client is also empowered with some affect regulation strategies.

3. The evaluation phase consists of the application of two evaluation instruments, the Subjective Units of Distress (SUD) and Validity of Cognition (VOC) scales, for assessing the negative cognition and the emotional response of the client to the investigated event.

4. The desensitization phase concerns the client’s emotional response, the evoked insights, and appropriate associations. In this phase, the client is bilaterally stimulated until the SUD level significantly decreases.

5. The installation phase aims at cognitive integration and restructuration. One essential aspect of this phase is the installation and strengthening of a more functional, positive cognition.

6. The sixth phase involves an analysis of the persisting body sensations which implies a mental body scan in order to identify any residual disturbance. Thus, the client will be able to process any unprocessed information.

7. The seventh phase includes discussions and prepares the client for the closure of the session. Its purpose is to bring the client to a state of equilibrium between the sessions.

8. The last phase is the re-evaluation of the progress at the beginning of each session.

Interaction with the user includes both audio and writing communication. Thus, the present research describes an EMDR virtual assistant system based on video, tactile, and audio actuators which includes an artificial intelligence chatbot that can be used in the autonomous treatment of traumatic memories.

With this background, the aim of our paper is to deliver an EMDR tool fully capable of assisting a user in performing efficient therapy and doing so without the intervention of a trained clinician. We developed a pilot study for testing our system, in which 31 participants were included. We found that our EMDR virtual system is efficient and can be used for the reduction of symptoms associated with PTSD and anxiety.

## 2. Related Work

Since EMDR therapy has proven to be efficient in the treatment of PTSD, recommended by the World Health Organization in 2013, it has also begun to be used in the treatment of resistant disorders, such as PTSD with comorbid psychosis [11], in which case it obtained a reduction of paranoid thoughts and allowed patients to reach remission in the psychotic disorder in a shorter period of time. For the treatment of borderline personality disorder [12], there had to be several adaptations made from the standard procedure. The same was done for people suffering from a narcissistic personality disorder [13] or comorbid bipolar I and II disorders [14].

EMDR therapy has been associated with technology since its earliest description. There was always an interest in the development of new devices to assist with the bilateral stimuli involved in the therapy process, whether these were devices designed to help in the visual phase of treatment, such as headsets and glasses, or devices designed to assist in the tactile therapy. Lately, with the advance in technology and the major development of mobile devices, there is interest in developing mobile applications that can provide the required treatment with as little involvement from a trained psychotherapist as possible. The literature on this topic is, however, scarce. We present below a brief summary of the existing studies and their major results.

In [15], Alulema Flores et al. present a prototype that can be used in EMDR therapy. It is a modular design containing three modules, each providing visual, auditory, and tactile sequences, integrated with an Android interface that controls the speed of the sequences, the type of sound, and the intensity of vibration of the tactile module. The software is intended for the use of the psychotherapist, who can either use a predefined routine or create a personalized program for the patient by controlling all three modules. The structure of the project consists of an Android device that controls the sound module and the IOIO for Android DEV-10748 motherboard [16] that controls both the visual and the tactile module. For each person, the application requires selecting a therapy type: the visual therapy lights the red LEDs on the glasses in different sequences, the touch therapy sends pulses to each hand at a time, and the sound therapy option plays from 10 different sounds in the left or right ear. Each type of therapy has an automatic and manual mode of functioning. In the manual mode, the psychotherapist can choose the frequency of the sequence (from 0 to 6 s) and also the side (left or right) that the sequence is displayed. In its conclusion, the paper [15] stated that the usage of the described system helped to reduce the processing time of patient cases and the duration of a therapy session, which decreased to 20–30 min per session. Also, when the system was used, it was observed that the level of physical exhaustion was reduced by 90 percent and mental exhaustion by 75 percent. However, no artificial intelligence chatbot was included in this system that could allow it to function fully autonomously—the intervention of the psychotherapist was still needed.

The system described by Jeffery D. Eastman in [17] is designed to help people that are suffering from trauma. It is based on the display of a series of light sequences that allows the user to self-direct an EMDR session. The implementation of the proposed method of treatment is based on the exchange of data between the patient’s computer, where the user completes a survey form after each therapy session, and the server application that responds with another adapted sequence of lights or in some cases even with an audio sequence. The self-directed session starts with the user logging in to the client application, which requests authentication and an initial light sequence from the server. The server returns a response based on the user’s history and personal data. Each treatment sequence is followed by a survey form containing a 1 to 10 scale evaluation of the discomfort level, which is taken into consideration for the next self-directed light session, and the light patterns are changed accordingly. As compared to the previous system, there are no tactile-based actuators in this system that can make the EMDR more efficient.

The invention presented in [18] by Gazit et al. refers to a system and a series of methods designed for remote EMDR therapy. The described system includes a therapist platform, a client platform, and an EMDR server which includes multiple processors, memory, a storage solution, and an operating system. The therapist platform presents a dashboard that allows the user to add client information such as personal data, preferences for bilateral stimulation, and notes regarding past therapy sessions, and controls different bilateral stimulation parameters. These parameters may refer to color, opacity, size, or background color in the case of video therapy; volume, sound effects, or the type of music for audio therapy; and for the tactile part, vibration intensity. For a better interpretation of the sessions’s the results of the session, the therapist’s computer has the function of displaying the level of correspondence between the client’s eye movement and the movement of the visual element on the screen. In this respect, an image processing algorithm running on the client’s platform can be used to correlate the movement of the visual bilateral stimulation and the client’s eye movement. Moreover, based on this implementation, the server may be configured to automatically modify the bilateral stimulation depending on the patient’s level of correspondence; for example, if the correspondence is better than 95%, the speed of the light display may be increased, while if it is less than 90%, the speed may decrease. This system does not include an intelligent chatbot.

In [19], Burgio et al. present a system and a method that can be used in EMDR therapy. The system is based on a headset, e.g., a pair of glasses, with dark lenses to block out the external light, a receiver that receives signals from an external device, a microprocessor, and data storage. EMDR therapy also implies auditory stimulation that can be provided by a device integrated with the headset and tactile stimulation which can be produced by a clothing article having incorporated a series of vibrating units over the torso. Also, in some embodiments, the stimulator may have a transceiver that can coordinate with the headset. Before every treatment, a diagnosis is performed by measuring the reactivity of each patient using a transducer assembly, with the role of determining the appropriate treatment schedule. An example of a treatment procedure may observe the following flow: first, the patient’s neuromuscular strength is measured with the transducer, then a specific treatment schedule is applied which may involve tactile stimulation, applying a gentle massage over the neural lymphatic points; visual stimulation, provided through the headset by changing the eye’s focus direction by displaying different light patterns; or auditory stimulation. During the treatment, the neuromuscular strength is remeasured. Then, depending on the weak signals that were determined, the patient may follow either a physical treatment, a mental treatment, or a chemical one. Next, the vibrating units and the headset are activated by the stimulator through an infrared signal and the treatment plan is applied. The process of the neuromuscular strength check is repeated, and an adapted treatment is followed again. This form of treatment that involves an automatic system is slightly different than the standard EMDR, but has proven to be cost-effective, enabling therapists to treat multiple patients at once. Also, the system can treat physical injuries, life accumulation stress disorder (LASD), and improve the patient’s overall mental and physical state.

As compared to all these studies, the system we propose has several improvements, such as the presence of an intelligent chatbot that can accompany the user during the EMDR intervention, which is developed in relation to the standardized procedures. Moreover, the presence of audio, video, and tactile-based actuators makes the EMDR system more efficient [20,21]. Table 1 describes these differences in relation to similar systems.

From a theoretical and practical point of view, this paper responds to some gaps reported in previous literature, having an important contribution [3]. Firstly, the proposed system is accompanied by an intelligent chatbot based on the protocol developed by F. Shapiro. In contrast, the other systems are based on a partial protocol or do not follow it at all and can only be used in the presence of a therapist. Other systems work remotely via an EMDR platform but still with the help of a person. As far as we know, there is no system similar to the one presented in this paper reported in the literature.

Secondly, because there is a constant need to test the potential effectiveness of self-administered EMDR therapy [3], this study aimed to address this need in testing the feasibility and efficiency of a virtual assistant for EMDR in reducing negative symptoms associated with traumatic memories.

## 3. Materials and Methods

### 3.1. Materials

The system offers a multi-actuator implementation of the EMDR protocol:

- Video stimulus using a rendered ball on a graphical display;
- Audio stimulus simulating a moving sound source;
- Tactile stimulation using vibration motors placed on the user using bracelets—stimuli are synchronized to maximize the effectiveness;
- A chatbot for communicating with the user according to the EMDR protocol, allowing the system to function autonomously, i.e., in the absence of a therapist.

**Figure 1:**
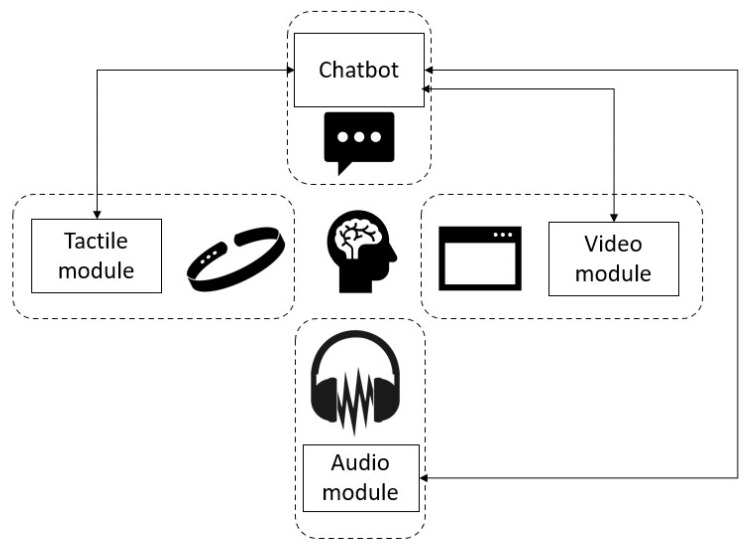
The EMDR system.

In Figure 1 we have a view of the general architecture of the system. We have chosen a component-based, highly modular approach so the system can be deployed in different configurations. The proposed system is composed of the following modules: video, audio, tactile, and chatbot, which can work independently or together, depending on the user preferences and deployment options.

**Figure A1:**
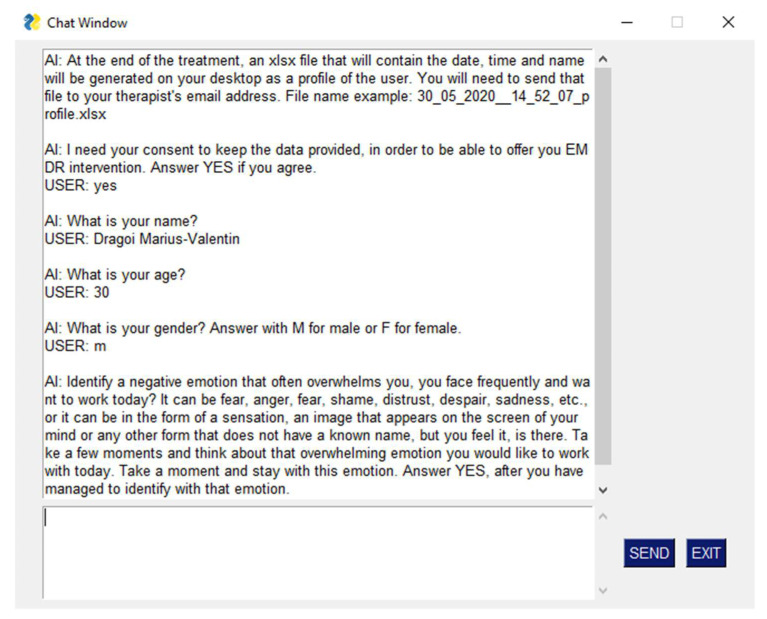
Chat window used for communication between the chatbot module and users.

*The chatbot module* is the central command module of the system, which coordinates and synchronizes all other modules, and allows users to interact with the system by text and audio (through the audio module). The chatbot is the kernel of the system because all processing decisions are made inside of it. Regarding user interaction, the module is programmed to describe each step of the treatment and asks a series of questions, according to the EMDR protocol, to evaluate the negative cognition and the emotional response of the user to a traumatic event. In the Figure A1 (see Appendix A), we present the interface of the EMDR system with the users and an example scenario. This chat window pairs the discussion between the artificial intelligence chatbot and users.

From the implementation point of view, we have developed the chatbot kernel in Python using different natural language processing algorithms, such as preprocessing, lemmatization and vectorization, and intelligent pattern matching based on cosine similarity. Chatbot intents are organized based on the stage of the protocol, with the purpose of giving contextual feedback to the user but also guiding her/him from stage to stage. The textual input from the user is preprocessed by removing stop words and lemmatization. Lemmatization is the process of finding out the dictionary form, or lemma, of a word, depending on the context. The WordNet lemmatizer was used for this task. Since most machine learning algorithms work on numeric input, the preprocessed text is then vectorized, by using a term frequency inverse document frequency vectorizer from Python.

The vectorized form is compared using an intelligent pattern match based on cosine similarity with the available patterns for a given intent. The highest match maps to a response outputted to the user.

**Figure 2:**
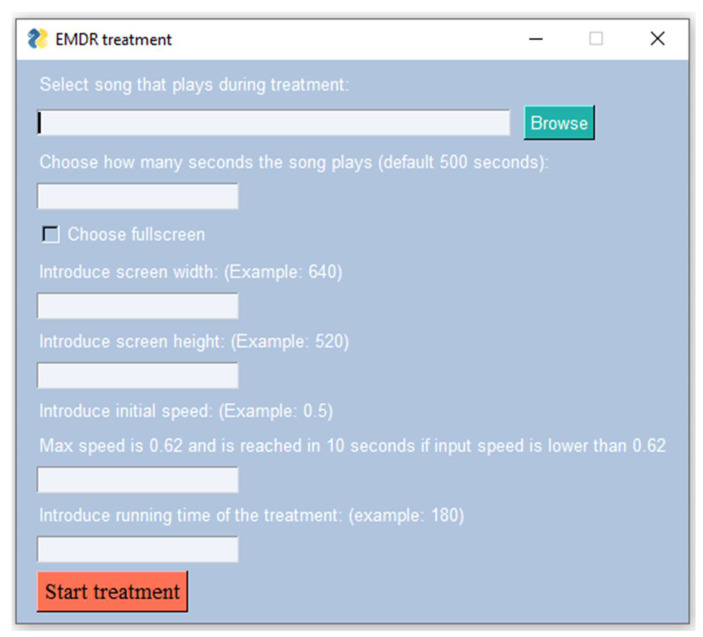
EMDR treatment user interface.

**Figure A2:**
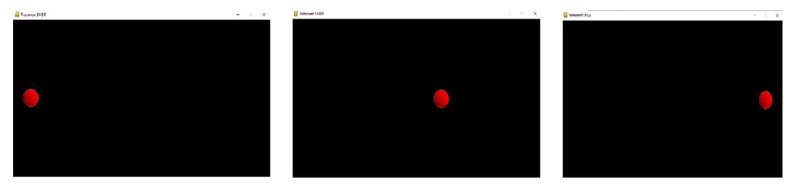
Movement of the ball during the EMDR session.

*The video module* is used in the desensitization phase to simulate the therapist’s hand movement by creating an output window that displays a red ball moving horizontally from one side of the screen to the other. The following parameters can be modified: window width and height and ball speed. Figure 2 presents the parameters of the motion, and Figure A2 (see Appendix A) displays the movement of the ball during the EMDR session.

The audio module has two responsibilities: firstly, to render audio for the chatbot interaction, the procedure descriptions, and the questions, and secondly, for the bilateral audio stimulation in the desensitization phase. This module can be deactivated for users with hearing impairments.

For the user interaction, the module can use pre-recorded audio clips by therapists but can also use a text-to-speech function to transform text input to audio output. This allows developers to easily add more interaction scenarios and provides more personalized and dynamic behavior, with text generated at runtime by the chatbot.

The audio bilateral stimulation is achieved by changing the sound balance from left to right and then back.

The *tactile module* is used in the desensitization phase to provide bilateral tactile stimulation, using vibration motors attached to the wrists of the patient, to complete the EMDR treatment experience, enhance the video and audio stimulations, and make the system usable by users with hearing and visual impairments.

**Figure 3:**
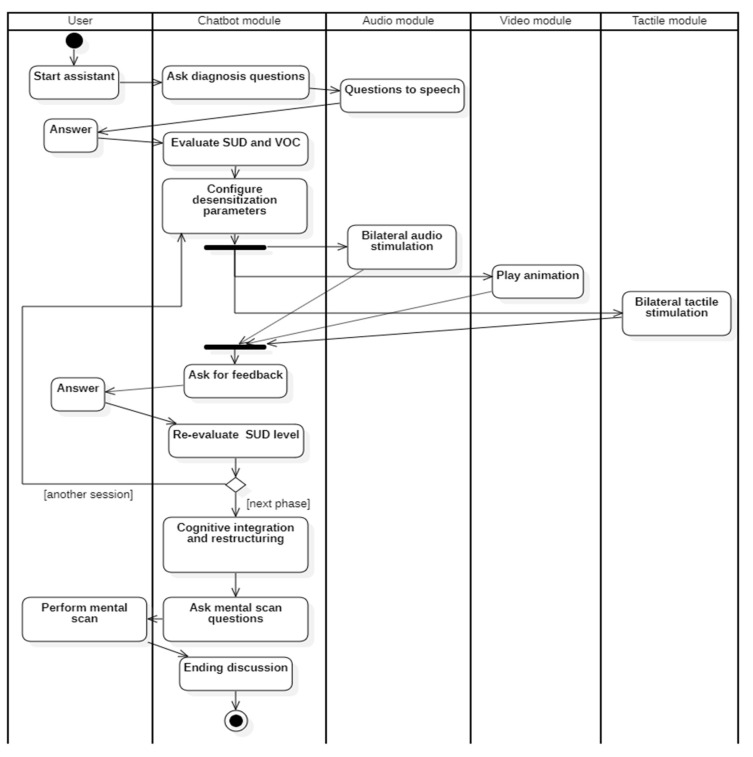
The overall flow of the system.

The overall flow of the entire system is presented in Figure 3 and is based on the EMDR protocol described in the Introduction section. The protocol is adapted so that the questions and evaluations typically carried out by the therapist are now implemented by the chatbot module (integrated with the audio module), while the desensitization phase is implemented in the audio, video, and tactile modules. We used a UML activity diagram to represent the actions conducted by each module in response to the patient input.

All modules were implemented in Python, with the chatbot module acting as a coordinator and also being the entry point of the application. Different libraries were used for the specific modules, such as PySimpleGui, time, sys, os for video module, gTTS text-to-audio library, playsound library for the audio module, NLTK, sklearn, and WordNet for the chatbot kernel.

**Figure 4:**
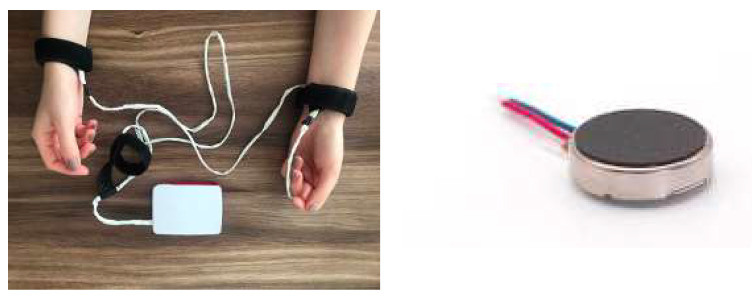
Raspberry Pi 4 with tactile module integrated and the vibration motor 1027 [22].

In Figure 4, the tactile module implements tactile stimulation by using two vibration motors pressed to the user’s wrists using bracelets. The motor used is the same as the one in [22], a small, lightweight flat vibration motor controlled by a Raspberry Pi board (or similar).

**Figure 5:**
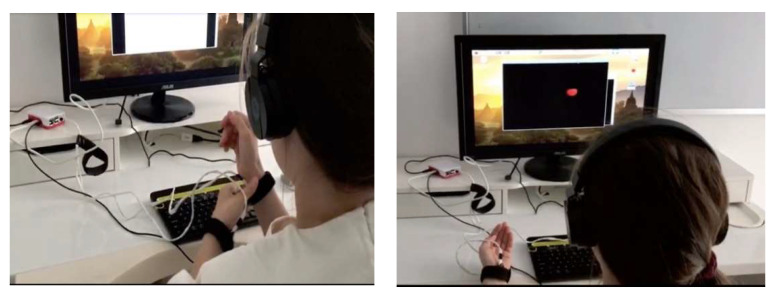
The participant is prepared for intervention. On the left side of the figure, we see how the user is fixing the actuators, while on the right side of the figure we see how the user is participating in an EMDR session.

The system can be entirely deployed on a Raspberry Pi (or similar) board, with an attached video and audio output, and the vibration motors attached to the RPI general-purpose I/O pins. If the selected configuration does not include the tactile module, then the system can also be deployed on a desktop/laptop computer. It is preferable to use headphones instead of speakers for bilateral audio stimulation. Figure 5 shows how somebody may use the EMDR system.

In the current pilot study, we tested the feasibility of an actuator-based EMDR virtual assistant system in replacing a trained clinician in the EMDR procedure and making the system capable of acting autonomously. Thus, we wanted to see if there were improvements regarding the cognitive and emotional response in relation to traumatic memories after the participants used the virtual assistant.

### 3.2. Recruitment

A total of 31 participants were included in the pilot study, 14 males and 17 females, with ages between 19 and 33 (M_age_ = 26.2, SD = 4.21). Given the nature of the study and given the fact that this pilot study is an initial step in exploring an innovative application, we followed the recommendation of Leon et al. [23] in recruiting and selecting participants, who emphasize that a pilot sample size is based on the pragmatics of recruitment and the necessity for examining the feasibility of the application. Participants were volunteers that responded to an online announcement and agreed to participate in a single session of intervention for reducing stress and anxiety associated with a potentially traumatic event from the past. In order to assess whether the participants were eligible in accordance with Shapiro’s recommendations, they were interviewed by two psychologists [10]. For inclusion in the study, participants needed to be 18–40 years of age, and to have at least medium scores on the Impact of Events Scale-Revised (IES-R) and the State–Trait Anxiety Inventory (STAI). Participants were excluded from the study if they scored very high on both measures, if they were suicidal, taking psychotropic medication, or had a diagnosed psychological condition. After the initial assessment, four participants were excluded (Table 2). All participants were informed about the study and signed a consent statement. The study was approved by the Committee of Ethics and Academic and Scientific Deontology, University of Medicine and Pharmacy of Craiova.

### 3.3. Procedure

The designed intervention consisted of four phases of bilateral stimulation (visual, auditive, and actuator-based). The first three phases were designed with the aim of reducing the intensity of emotions and beliefs associated with the traumatic event. The fourth phase was introduced to install a positive belief about that event. In addition to visual stimulation, audio and tactile stimulation were used simultaneously, as auditory, and tactile stimuli have been shown to enhance the effect and increase the effectiveness of EMDR intervention [20,21].

The procedure was applied as follows. The participants were informed about the EMDR therapy and instructed on how to use the application. Participants were invited to think about a traumatic event from the past that caused them discomfort, where they failed to overcome it. After accessing the traumatic memory, the IES-R and STAI instruments were applied in the pre-test condition. After the discussion with a specialist, and after the completion of the instruments on the pre-test assessment phase, the participants self-administered the intervention through the application. After the initial assessment, we eliminated four participants because their scores on the IES-R and STAI were very high, and they needed specialized professional treatment. In Table 3 we can see the description of the applied protocol in detail.

**Figure 6:**
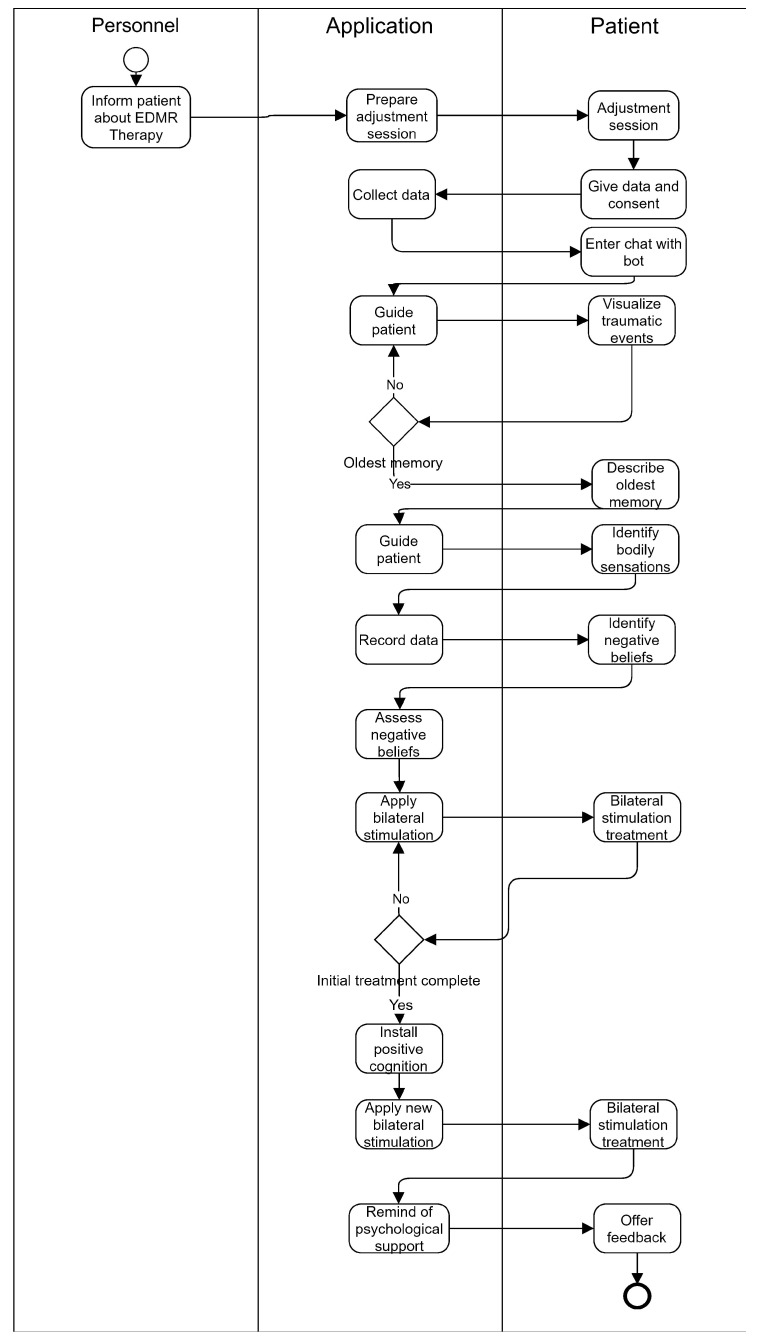
The representation of the applied application protocol.

The detailed steps of the application protocol and their interaction on multiple levels are visually represented in Figure 6.

### 3.4. Instruments

#### 3.4.1. Primary Outcome Measures

Impact of Event Scale-Revised (IES-R). The IES-R is a 22-item self-report questionnaire used to assess subjective distress caused by a recent traumatic event or a specific one. The IES-R items are rated on a 5-point scale ranging from 0 (“not at all”) to 4 (“extremely”) and related to symptoms associated with PTSD. The IES-R scale can have a total raw score ranging from 0 to 88 and a raw score for three subscales: Intrusion, Avoidance, and Hyperarousal subscales. For our study, we calculated the total score in order to assess the severity of the symptoms [24,25]. The scale has good psychometric properties being validated for the Romanian population and is one of the most used instruments for assessing traumatic events [26,27,28]. In this study, Cronbach’s alpha varied from 0.87 in the pre-intervention phase to 0.72 for the post-intervention condition.

The State–Trait Anxiety Inventory (STAI) is a 40-item self-reported instrument used to measure trait and state anxiety and was designed by Spielberger et al. [29]. The scale consists of two separate subscales each of 20 items that measure the state factor and the trait one. In our study, we used the first subscale which measures the state factor because this scale can be used to measure the level of anxiety regarding a situation, event, or traumatic memory from the past. All items are rated on a 4-point scale ranging from “Almost Never” to “Almost Always”, with higher scores indicating higher levels of anxiety. The scale was validated for the Romanian population [30]. In our study, Cronbach’s alpha was 0.88 in the pre-intervention condition, and 0.71 for the post-intervention condition.

#### 3.4.2. Secondary Outcome Measures

Apart from these primary outcome measures, we used other scales that are part of the standardized intervention procedures in EMDR [10].

Subjective Units of Disturbance (SUD) scale is a single-item measure and self-reporting scale used for measuring the level of subjective distress and intensity of the traumatic memory with answers ranging from 0 to 10, where 0 refers to a lack of disturbances and 10 to the worst possible.

The Validity of Cognition (VOC) scale is a single-item measure and self-report scale and shows the level of belief in a positive or a negative cognition on a scale ranging from 1 to 7, where a score of 1 means a lack of belief in a particular cognition, and a score of 7 means a full belief in it.

## 4. Results

### 4.1. Primary Outcome Measures

The results provided in Table 4 and Table 5 show that there was a significant difference in scores for the pre-test condition (M = 39.8, SD = 14.07) and post-test (M = 13.7, SD = 5.7) measured with the IES-R scale: t (30) = 10.5, *p* < 0.001, and a large effect size (d = 1.89).

Also, there was a significant difference in scores for the pre-test condition (M = 56.7, SD = 8.3) and post-test (M = 33.8, SD = 4.6) regarding anxiety scores: t (30) = 11.7, *p* < 0.001, and a large effect size (d = 2.11) of intervention in reducing anxiety associated with the traumatic event. In other words, the intervention through the virtual assistant system for EMDR had a significant effect and contributed to reducing the distress associated with the traumatic event, and reduced the anxiety associated with it.

### 4.2. Secondary Outcome Measures

We used the VOC and SUD scales in order to assess whether there were improvements in the EMDR procedure on the negative cognitions and emotions associated with the traumatic event. The results indicate a significant increase in VOC scores: t (30) = 14.4, *p* < 0.001, which reflects a positive increase of positive cognition regarding the traumatic event (Table 6). Also, we notice that there was a significant pre- to post-intervention reduction in scores for SUD: t (30) = 75.9, *p* < 0.001 (Table 5). Table 7 also shows that the higher scores decreased significantly at the end of the intervention. These results indicate that the self-administered protocol has good potential and is efficient for reducing the negative cognitions and emotions associated with the traumatic memory even in the absence of the human factor.

## 5. Discussion

The goal of this study was to develop an EMDR virtual assistant capable of guiding a user in performing an efficient therapy with minimal intervention from a trained clinician. Given the fact that the study is a pilot one, the process was supervised by trained clinicians who did not intervene in the process but were prepared to do so if needed.

The results suggest the efficiency of self-administered EMDR through the developed system pre- and post-intervention. Our study showed a statistically significant reduction of symptoms associated with PTSD and anxiety. Also, the self-administration of treatment using bilateral actuator, visual, and tactile stimulation appears to produce a significant decrease in symptoms associated with traumatic memories. The intervention contributes both to the reduction of negative affections associated with traumatic events and helps in changing these cognitions with more functional ones.

Compared to other systems that offer the possibility of EMDR interventions, such as the one proposed by Aleluma Flores et al. [15], our virtual system is more autonomous, being developed following the EMDR protocol, and the presence of a clinician is not mandatory. In addition, the main difference from other systems [17,18,19] lies in the usefulness of the intelligent chatbot together with the multi-actuator implementation that can have an impact on the efficiency of the intervention.

Our results are in line with interventions that used traditional EMDR [8,31]. Even if the intervention involved only one session, our findings are similar to those of previous research, which showed that even after a single session of EMDR, symptoms decreased significantly, effects of the intervention being constant after follow-up [32,33,34,35,36,37]. Moreover, this decrease in terms of symptoms was also found in other research where participants were treated remotely and the intervention was self-administered [3,38].

In explaining our results, as noted by Kutz et al. [37], we must take into consideration two main variables: the former exposure of participants to trauma and the presence of some risk factors that can influence the effect of the intervention [35]. In this respect, those who are exposed to a single traumatic event and who have experienced moderate symptoms associated with PTSD or anxiety are more prone to have a rapid response in terms of recovery, compared to those who have a history of risk factors or pre-existing vulnerabilities such as multiple exposures to significant trauma [37].

Also, it is important to note that in relation to the virtual assisted treatment, the participants were curious about how the intervention would proceed, while some were interested in the possibility of working through their traumatic memories without exposing themselves to another person. Therefore, our system could be very useful in the context generated by COVID-19, given that most of the activities are developed in the online environment, and the incidence of depression, anxiety, insomnia, and PTSD has increased substantially [39]. Because of the impact that COVID-19 has on individuals and in the context of increasing demands for remote intervention [40], the possibility of using such a system complements the lack of human resources and at the same time makes psychological intervention possible where, because of the restriction measures, face-to-face interaction with a specialist is not possible. Moreover, our system is suitable for cases confronting mild psychological distress, which do not require the presence of a specialist. Thus, existing human resources can concentrate on those cases that require special attention.

Based on the results from this study, we can state that having a system which offers an autonomous EMDR intervention is a clear possibility. The advantages include reduced costs and increased accessibility. However, it raises several questions about whether the technology can replace the human presence entirely. We are aware of the fact that there are some risks associated with this intervention, such as increased short-term distress for some participants. Still, studies have shown that such risks are minor and are based on anecdotal evidence [3]. We have improved our system by taking into account the recommendation for designing safe, affordable, and easy-to-use interventions for self-help that have the potential to be used by many people [41]. However, we consider that any intervention, whether autonomous or not, must be carried out in the first phase under the supervision of a trained clinician and in compliance with the established protocol until large-scale testing.

Participants did not report any problems in their interactions with the system. The usability of the system was considered good and whenever needed, explanations were given to the participants on how to use the system. Overall, the experience of the users in this respect—usability—was ranked as good.

However, the results of our present research must be interpreted in the context of the pilot study that we have carried out, which is based on a limited number of participants and consequently has limitations in terms of generalizability. Even so, this is a first step in addressing the need that has been reported in previous studies regarding the development of self-administered EMDR therapy that is developed in accordance with established protocols and underscores the need for more extensive research. We consider it as another step in studying the effectiveness of remotely administered interventions and a starting point for developing some clinical trials using the proposed system.

## 6. Conclusions

In this paper, we described an EMDR virtual system based on video, tactile, and audio actuators used to treat anxiety, distress, and negative emotions associated with traumatic memories. Based on the results obtained, an autonomous EMDR intervention is a clear possibility.

Our study evidences the efficacy of an EMDR intervention for those who cannot have access to immediate psychological support, thus enabling their autonomy. Moreover, the established protocol was tested and positively confirmed in providing the necessary guidance for an efficient administration. As a result, it could be considered as a tool of self-support for people experiencing mild symptoms of PTSD.

Moreover, one of the key findings of this study refers to the efficiency of such a system that offers the possibility of self-administration of an EMDR intervention, thus contributing to a limited body of research in this field [3]. Even if there are some developments and applications for EMDR therapy, the majority of the systems are not autonomous and require the permanent intervention of a clinician or are used only as a tool in treatment [38,42]. Compared to other systems, the unique advantages of the present system are as follows:

- The presence of an AI-driven chatbot, following the eight essential phases of EMDR [10], which can be further enhanced with new dialogue options and evaluation strategies;
- The ability to customize the interaction based on the patient’s characteristics. For example, using different stimulation methods (visual, audio, tactile) makes the EMDR procedure accessible for people with disabilities (severe visual impairment or even blindness, hearing loss);
- The possibility of applying EMDR treatments in a fully unsupervised manner, using the aforementioned chatbot, i.e., the assistance of a trained clinician is not required;

Future work will include adding web-based functionality to the system to make it possible to be used by the public. Adding intelligent dialog robots, such as Siri, can realize autonomous communication and at the same time can accompany the user in accommodating the intervention. Moreover, the use of an automated system has the potential to enable future studies comparing EMDR with alternative treatments using sham stimulation, which could be valuable in helping to elucidate the efficacy of the bilateral stimulation component of EMDR at a more general level.

## Figures and Tables

**Table 1:** Comparison with similar systems in the literature.

Paper	Advantages	Disadvantages	Differences between Our System and Related Work
Aleluma Flores et al. [15]	Uses visual, tactile, and auditory therapy. Can be customized.	Implements only the desensitization phase in the EMDR protocol. Used only by psychotherapists/clinicians. The presence of a trained clinician is mandatory, cannot be used independently.	Presence of intelligent chatbot which implements the preparation, evaluation, and ending phases in the EMDR protocol. The system has the potential to be autonomous, the presence of a trained clinician not being mandatory.
Jeffrey D. Eastman [17]	Visual stimulation can be dynamically adjusted based on feedback from the user.	Only visual and auditory stimuli. Audio is mostly used for playing relaxation sounds, very little information is given about bilateral audio stimulation. Only patent, not validated.	Presence of intelligent chatbot which implements the preparation, evaluation, and ending phases in the EMDR protocol. Audio, video, and tactile-based actuators that make the EMDR system more efficient.
Gazit et al. [18]	Selection of the intensity of stimuli.	No intelligent chatbot. The system is designed to intermediate the relation between client and clinician and can be used remotely but requires the intervention of a therapist for setting the parameters.	The proposed system has the potential to be autonomous.
Burgio et al. [19]	Opaque glasses that block light from reaching the eyes and thus prevent simultaneous exposure to other visual stimuli. Uses chemical stimulation.	The system is dependent on the presence of the therapist Different from the standardized EMDR procedure Only patent, not validated.	The proposed system includes an intelligent chatbot offering autonomy. Designed in relation to standardized EMDR procedures.

**Table 2:** Sample characteristics.

Characteristics	Category	*n*	%
Age (years)	Mean ± standard deviation	26.2 ± 4.21	
Gender	Male	14	45.2
	Female	17	54.8
Education	High school diploma	4	12.9
	College degree	10	32.3
	Master’s degree	15	28.4
	Doctorate (Ph.D)	2	6.5
Health condition	No treatment in the last 6 months	23	74.2
	Under treatment in the last 6 months	8	25.8
Months since the traumatic event	Less than 6 months	1	3.2
	Between 6 and 12 months	13	41.9
	Between 12 and 24 months	6	19.4
	Between 24 and 36 months	7	22.6
	More than 36 months	4	12.9
Criteria for inclusion	(18–40 years of age; at least medium scores on IES-R and STAI)	31	88.5
Criteria for exclusion	(Scored very high on both measures; suicidal; taking psychotropic medication; diagnosed psychological condition)	4	11.6

**Table 3:** Application protocol description.

The Phase of the Intervention	Description
1. Introduction	Participants were informed about EMDR therapy and how bilateral sensory stimulation helps process traumatic memories. It was mentioned that they could withdraw at any time if they were not comfortable with the procedure. At the same time, the participants went through an adjustment session with bilateral stimulations.
2. Demographic info	The application requested demographic information (gender and age) and the agreement for participation.
3. Preparation for accessing the traumatic event	Through intelligent chatbot guiding, the participant was led to access overwhelming emotions that sometimes interfere with their usual activity and primary events recorded in memory associated with overwhelming emotions. The participant was also guided to visualize that event mentally as a movie scene. Once the oldest memory associated with those emotions was identified, the participant was asked to describe it briefly.
4. Body scanning	The participant was intelligently guided to identify the bodily sensations associated with that event (tension, tremor, cold, heat, pressure, or sounds). The information obtained was noted and recorded by the application.
5. Identification of negative beliefs	Identification of negative beliefs (about oneself, others, the world, or life in general) caused by the traumatic event.
6. Emotions and cognitions intensity assessment	Assessment of the intensity of negative cognitions/beliefs and emotions associated with that event (VOC and SUD scale)
7. Treatment phase	Application of bilateral stimulation (audio, video, and sensory) in several sessions of four minutes each and assessment of the intensity of cognitions and emotions after each session of bilateral stimulation done through the guidance of the chatbot.
8. Positive cognition identification and installing	Identifying and installing positive cognition through a new session of bilateral stimulation.
9. Closure	The intelligent chatbot reminds the participant that they can benefit from psychological support.
10. Feedback	The participant can share their impressions related to the experience of using the application.

**Table 4:** Paired sample *t*-test—primary outcome measures.

					95% CI For Cohen’s D	
	t	df	*p*	Cohen’s d	Lower	Upper
IES-R Pre-, IES-R Post-	10.521	30	<0.001	1.890	1.292	2.476
STAI Pre-, STAI Post-	11.759	30	<0.001	2.112	1.469	2.744

**Table 5:** Descriptive statistics of scores (pre- and post-intervention).

	N	Mean	SD	SE
IES-R Pre-	31	39.806	14.077	2.528
IES-R Post-	31	13.742	5.785	1.039
STAI Pre-	31	56.774	8.330	1.496
STAI Post-	31	33.839	4.620	0.830

**Table 6:** Paired samples *t*-test—secondary outcome measures (pre- and post- assessment).

	t	df	*p*
VOC Pre-, VOC Post -	14.471	30	<0.001
SUD Pre-, SUD Post -	75.904	30	<0.001

**Table 7:** Pre- and post-intervention comparison of secondary outcome measure.

	N	Mean	SD	SE
VOC Pre-	31	2.968	0.657	0.118
VOC Post-	31	6.097	0.887	0.156
SUD Pre-	31	9.161	0.735	0.132
SUD Post-	31	0.774	0.717	0.129

## Data Availability

The data presented in this study are available upon request from the corresponding author.
